# Supplementary material for: Rapid turnover of life-cycle-related genes in the brown algae
Source: Genome Biol. 2019 Feb 14;20:35. doi: 10.1186/s13059-019-1630-6 (PMC6374913; doi:10.1186/s13059-019-1630-6)
Supplement: Supplementary file 3 — Protein alignments, corresponding Gblocks html file and CDS sequences for the lineage conserved gametophyte-biased genes. (ZIP 2109 kb) [file 13059_2019_1630_MOESM3_ESM.zip › Con_GA_alignments/Ec-06_002540.aa_ali.fasta-gb.htm]

Ec-06\_002540.aa.fasta.trans.aa\_ali.fasta


## Gblocks 0.91b Results

Processed file: **Ec-06\_002540.aa.fasta.trans.aa\_ali.fasta**  
Number of sequences: **2**  
Alignment assumed to be: **Protein**  
New number of positions: **535** (selected positions are underlined in blue)

```
                         10        20        30        40        50        60
                 =========+=========+=========+=========+=========+=========+
Ec-06_002540     MTVESRSSAATVTETQGATIPFERSNDVIREGAPPSDAADDALAREDNHTGTEMDHTVAH
MSTRG.20423.1    ------------------------------------------------------------
                                                                             


                         70        80        90       100       110       120
                 =========+=========+=========+=========+=========+=========+
Ec-06_002540     TVLEAPASSACLELLATNAKPVDDNHQPQHSQTPEHLAPDKPEGISLLGPEATEQPYCAK
MSTRG.20423.1    ------------------------------------------------------------
                                                                             


                        130       140       150       160       170       180
                 =========+=========+=========+=========+=========+=========+
Ec-06_002540     TSGPGAGQESEGEGAARSLASAASPSDSGTAGFEALPSATTGTSDEKQQEEQKLSDVDSV
MSTRG.20423.1    ------------------------------------------------------------
                                                                             


                        190       200       210       220       230       240
                 =========+=========+=========+=========+=========+=========+
Ec-06_002540     DSCRDEEVAASDRDDGAGPPSEGPCYRESAAGDSETLSPAVESEEDDSVAQKDVSAAQKR
MSTRG.20423.1    ------------------------------------------------------------
                                                                             


                        250       260       270       280       290       300
                 =========+=========+=========+=========+=========+=========+
Ec-06_002540     CDSVREVSAGADSMGTKQSPASGNDESARSTHITSLDGVEEMHPLQPPTRHDLQTSCGCA
MSTRG.20423.1    ------------------------------------------------------------
                                                                             


                        310       320       330       340       350       360
                 =========+=========+=========+=========+=========+=========+
Ec-06_002540     SPTVEAVTVGKDSVQYCGGGDENLRGFLLVSGDAQHFVEDVTSSSLAAFSRDKLRRPELA
MSTRG.20423.1    ------------------------------------------------------------
                                                                             


                        370       380       390       400       410       420
                 =========+=========+=========+=========+=========+=========+
Ec-06_002540     VEMILPGAANASRETPPMGRGVNSEEVTRPLPDDSQHNEPAPSPDSSLCKPPLVEITEGL
MSTRG.20423.1    ------------------------------------------------------------
                                                                             


                        430       440       450       460       470       480
                 =========+=========+=========+=========+=========+=========+
Ec-06_002540     ISRVFEVVAAKMAAAAAEASTHVAVPEVATTAERLELKILPEVDFPCDSPSASEGMPADG
MSTRG.20423.1    ------------------------------------------------------------
                                                                             


                        490       500       510       520       530       540
                 =========+=========+=========+=========+=========+=========+
Ec-06_002540     AIQPPDEARPDFGQLSLGSESPAKQADVMDDDRKSTGVHAAEVSGGTGDDQEEATAPATP
MSTRG.20423.1    ------------------------------------------------------------
                                                                             


                        550       560       570       580       590       600
                 =========+=========+=========+=========+=========+=========+
Ec-06_002540     ATANNTPPQPHSSCSLPGTVVEEYGLHEIPRLLAAEVVTAAIAAVMPISSSACEPASVTT
MSTRG.20423.1    ------------------------------------------------------------
                                                                             


                        610       620       630       640       650       660
                 =========+=========+=========+=========+=========+=========+
Ec-06_002540     APPPSDSGAEAGTETTVKADSLTAGQDPASDLAASSTIVEQDSAEACPAVGDDLVGRVEN
MSTRG.20423.1    ------------------------------------------------------------
                                                                             


                        670       680       690       700       710       720
                 =========+=========+=========+=========+=========+=========+
Ec-06_002540     GLAVGQESASDLALAARSIIVEQESVEERESCPDDGDDLVRSVEENPCGDGPRSPTPPST
MSTRG.20423.1    ------------------------------------------------------------
                                                                             


                        730       740       750       760       770       780
                 =========+=========+=========+=========+=========+=========+
Ec-06_002540     REGEGTSTIDPEPEREAPSRAESTAGGDRQPRHDSRSAEALLYLTEAGSRAKSRQQRSEE
MSTRG.20423.1    ------------------------------------------------------------
                                                                             


                        790       800       810       820       830       840
                 =========+=========+=========+=========+=========+=========+
Ec-06_002540     IRVDASSWLRDAGGRALAAQGEGDAEGLEHAVVAEYVTTTLQRVVAEEAVAVAGVAPVSP
MSTRG.20423.1    ------------------------------------------------------------
                                                                             


                        850       860       870       880       890       900
                 =========+=========+=========+=========+=========+=========+
Ec-06_002540     SACAPETKTGDQATPRVPPTLEPGTPEGDRFSNTGPGLHSEAPCFVRSAADGDKSPAPGD
MSTRG.20423.1    ------------------------------------------------------------
                                                                             


                        910       920       930       940       950       960
                 =========+=========+=========+=========+=========+=========+
Ec-06_002540     IDASTCLAEDGSRAKSLRENVGDNRVDTSPRLRDTEERTNSPQSVDQAALLERAVVTDYL
MSTRG.20423.1    ------------------------------------------------------------
                                                                             


                        970       980       990      1000      1010      1020
                 =========+=========+=========+=========+=========+=========+
Ec-06_002540     SGALTRAVEAEAATADAALDASSSEPSNGGMMGKDQATPGAPYPRGDTEDAINVAEIGAK
MSTRG.20423.1    ------------------------------------------------------------
                                                                             


                       1030      1040      1050      1060      1070      1080
                 =========+=========+=========+=========+=========+=========+
Ec-06_002540     ADDEISSGEDPVPELAMHSPVCEATATGEQESPDHGDTSPKDGDDQSDKANKNGSDGLQL
MSTRG.20423.1    ------------------------------------------------------VEALSL
                                                                             


                       1090      1100      1110      1120      1130      1140
                 =========+=========+=========+=========+=========+=========+
Ec-06_002540     PTPLCTPEEEGPSTDKSEIKPEVPSNAVLTADGSGSPAPRSTEALVYLTEAGERAKSLQE
MSTRG.20423.1    P-----------ARTSKDLR----------------------EAAEWLLLVGGRAKSAQD
                                                           ##################


                       1150      1160      1170      1180      1190      1200
                 =========+=========+=========+=========+=========+=========+
Ec-06_002540     GSEKARLDASLWLRDTAGKALSRESVATADPSLDTPPGTVTAELAAAAAAASPPSTACTP
MSTRG.20423.1    RVEKARINASSWLREKGGRALS--------------------------------------
                 ######################                                      


                       1210      1220      1230      1240      1250      1260
                 =========+=========+=========+=========+=========+=========+
Ec-06_002540     PAETTVDAQSVQAASTLSPTCDPGSPEKQERISDADQEPGQEKPSGVVSATVGDGQSPSR
MSTRG.20423.1    ------------------------------------------------------------
                                                                             


                       1270      1280      1290      1300      1310      1320
                 =========+=========+=========+=========+=========+=========+
Ec-06_002540     GSTEGLTCLEEGGEREKESSEGRGVDGSSFVRDTEGRTPSVEDKKQAARAECVVVEEDLS
MSTRG.20423.1    ----------------------------------------LSDREQARRSECAA------
                                                           ##########        


                       1330      1340      1350      1360      1370      1380
                 =========+=========+=========+=========+=========+=========+
Ec-06_002540     ETMAKDVGVEVAATATTTAAPSTPGASAPASAGRDKATLGASAPRDPGAADVAENAVEAD
MSTRG.20423.1    ------------------------------------------------------------
                                                                             


                       1390      1400      1410      1420      1430      1440
                 =========+=========+=========+=========+=========+=========+
Ec-06_002540     DVTTTVRQDFESALRFSIVEAMAVVGQGSAGQRESSEKGGRCVIDTSRERPGSPTPPGTP
MSTRG.20423.1    -------------------------WLGSVGGR---------------------------
                                                                             


                       1450      1460      1470      1480      1490      1500
                 =========+=========+=========+=========+=========+=========+
Ec-06_002540     EEDTPRTSSLGLDPEVTFNPDTDPADRGQPPPPQPAEASSACPAEVGLREEALREASGWL
MSTRG.20423.1    ------------------------------------------------------------
                                                                             


                       1510      1520      1530      1540      1550      1560
                 =========+=========+=========+=========+=========+=========+
Ec-06_002540     LLAGGRSAQDRAERAREEASSWLRDTGGRAVSLQDRAEGVRSGAAAWLAARGASELEQTA
MSTRG.20423.1    ----AKSAQGRADKAREDASSWLRSAGLGAVSLQDRAEGARSDAAAWLKSKAISELERTG
                       ######################################################


                       1570      1580      1590      1600      1610      1620
                 =========+=========+=========+=========+=========+=========+
Ec-06_002540     PTTRRAEDATAEASAAEVKGEEDGQGTGESKSLQGPPTQDGSRPGSPFASSPSQPEKPSA
MSTRG.20423.1    PVEGRVE-VLAEASLALRQGKPEAEDTG-------HPFKCGRLNESPVTASDSPHTGYHP
                 #######   ##################        #################       


                       1630      1640      1650      1660      1670      1680
                 =========+=========+=========+=========+=========+=========+
Ec-06_002540     ATAETEDVVARAADQETTREAPSHNAEEAVTAKECFPTVGKDDRAENINNTSTPSPAHDQ
MSTRG.20423.1    NITATEVVAPGSSGRDIRQDTPPGDNEAVQPLERPSDDSTSSHVHPTVTNDASILSPPRQ
                                                                            #


                       1690      1700      1710      1720      1730      1740
                 =========+=========+=========+=========+=========+=========+
Ec-06_002540     TLNPAESAAAAAAAAPVAITTANTAAVYPTAQLTIGRTASSDRTAPVPWQEPATTPLDRG
MSTRG.20423.1    APSLVEGEAAATSTTPTAADATSTDF-----EQPPGRTASTDRTARAPWLEPVTSPL---
                 ########################           ######################   


                       1750      1760      1770      1780      1790      1800
                 =========+=========+=========+=========+=========+=========+
Ec-06_002540     GSFAPTAAGPEDLGRMLDAGEKEWSRVSLSRTPPFDALNSPSLPQRYAPIRGRPRPLPP-
MSTRG.20423.1    ---VSTAAGTEDFGRMLGAGEKEWARVSLSRTPPFDALNSPSLPVRYAPVRGRPRPLPTD
                      #####################################################  


                       1810      1820      1830      1840      1850      1860
                 =========+=========+=========+=========+=========+=========+
Ec-06_002540     -------------------------EAKAEAVAGAGAGVGPNADGPGW--FFAPPDQMAP
MSTRG.20423.1    AEVFETGGAGAAAVAAGADPAIPAGEAMHRRVSGGGAGAAHNADGSGWVEFFAPPDQMAP
                                          #######################  ##########


                       1870      1880      1890      1900      1910      1920
                 =========+=========+=========+=========+=========+=========+
Ec-06_002540     KGDAYRGSDSFHSADASASTTVRGDSS--GSFVS-----PSQGGSGGTDDGRRPRRRDNG
MSTRG.20423.1    RGDAYRGSDGCGSAEASASTTARADSSSAGSLTSSLAGHPRRRERDGSDDPHRRKQRDDE
                 ###########################            #####################


                       1930      1940      1950      1960      1970      1980
                 =========+=========+=========+=========+=========+=========+
Ec-06_002540     GSGGD-HASTRCFDG-------GGGGGGVWEETTLVDGLGDAINPAREAQCRMMNRAAVA
MSTRG.20423.1    DREGDGHGNDRAWRFSGDVGVDGRGVGRRSSEEAWGETLGEVSHPAGEACGRMVDRISVA
                 #####                 ######################################


                       1990      2000      2010      2020      2030      2040
                 =========+=========+=========+=========+=========+=========+
Ec-06_002540     GEEGKAHLGRRWEETGTP-----------SLGSRNTRGSGNRWHKKVEQSRQYLRRWEVF
MSTRG.20423.1    SR-------GRWDETGSFSPASREVRGNNGGVSGHRAGGGKRWHKKAEQSRQYMRRWEAF
                                                 ############################


                       2050      2060      2070      2080      2090      2100
                 =========+=========+=========+=========+=========+=========+
Ec-06_002540     LSRAHRHFDGEADVRKRMSVIRRAHPDVSNEVAFVALAQSRGRSSEAAAVLHLPRAKDEA
MSTRG.20423.1    LGRTHRYFDGEADVRKRMSLVRRAHPDVSNEVAYVALAQSRGRSSEAAAVLHLPRAKDEA
                 ############################################################


                       2110      2120      2130      2140      2150      2160
                 =========+=========+=========+=========+=========+=========+
Ec-06_002540     ALVAMMLDVTAFIGLAREAAERRRRSRQLEHQRRRMETVAGGAAKRPAHHHQGKGEHRQP
MSTRG.20423.1    AMVAMMLDVTSFIHLARQASERRRRFRHLERERR-------------AIAVGGRGEARGP
                 ##################################             #############


                       2170      2180      2190      2200      2210      2220
                 =========+=========+=========+=========+=========+=========+
Ec-06_002540     SWRQHQRQQEEWYSHPEDHRRDSAEDKQRLNNYIWEQQQQRQKQGEERRSGHEWARDDAS
MSTRG.20423.1    ------------------FRRKGGADGMLPQ-----------------------------
                                                                             


                       2230      2240      2250      2260      2270      2280
                 =========+=========+=========+=========+=========+=========+
Ec-06_002540     TGVIGDAAENNWHSQRNKQDYGAEPISAGGNAGRSRGEAQSRRSDSAPSLLPPIEGWLTE
MSTRG.20423.1    ------------------------------------------------------------
                                                                             


                       2290      2300      2310      2320      2330      2340
                 =========+=========+=========+=========+=========+=========+
Ec-06_002540     GTPPSPNSLKTVLSTSTSVAVGVPSGFGLASVAGVASKGEGGKIVRVRSSKSTSRELRAM
MSTRG.20423.1    ------------------------------------------------SPTSACQEL---
                                                                             


                       2350      2360      2370      2380      2390      2400
                 =========+=========+=========+=========+=========+=========+
Ec-06_002540     LRARARWQSQRMAEMATPGSTEQRQQQGWGGSGLDALAERFLRETDTLDRDSKDLDTRLR
MSTRG.20423.1    --GEERW-----------------------------------------------------
                                                                             


                       2410      2420      2430      2440      2450      2460
                 =========+=========+=========+=========+=========+=========+
Ec-06_002540     GRQKNKRGPSKLRGPHNDGGGGRGERRVFGNGGGDAAWRGGDSGDGVGGRGGGRGRGGSG
MSTRG.20423.1    ------------------SGGGR-------EGGVTDA--EGYKGDMATDKNGCRLRLRQE
                                                         ################    


                       2470      2480      2490      2500      2510      2520
                 =========+=========+=========+=========+=========+=========+
Ec-06_002540     RRRPSLSSSSSSSSMFLASVNRDPNAPFMSLETPAAVVVVDGGSGVCSTSSFAAAAVSDP
MSTRG.20423.1    ------HRRREISSELLPVEARKRNA----------------------------------
                             ##############                                  


                       2530      2540      2550
                 =========+=========+=========+=======
Ec-06_002540     ATAAAASAASCAVEADGGEDRESCREQGYFTQAGGWR
MSTRG.20423.1    -------------------NRRSSSSSSX--------
```

```
Parameters used
Minimum Number Of Sequences For A Conserved Position: 2
Minimum Number Of Sequences For A Flanking Position: 2
Maximum Number Of Contiguous Nonconserved Positions: 8
Minimum Length Of A Block: 10
Allowed Gap Positions: None
Use Similarity Matrices: Yes
```

```
Flank positions of the 16 selected block(s)
Flanks: [1123  1162]  [1303  1312]  [1507  1567]  [1571  1588]  [1597  1613]  [1680  1704]  [1716  1737]  [1746  1798]  [1826  1848]  [1851  1887]  [1900  1925]  [1943  1980]  [2013  2134]  [2148  2160]  [2441  2456]  [2473  2486]  

New number of positions in Ec-06_002540.aa.fasta.trans.aa_ali.fasta-gb:  535  (20% of the original 2557 positions)
```
